# Proteins adopt functionally active conformations after type III secretion

**DOI:** 10.1186/s12934-016-0606-4

**Published:** 2016-12-23

**Authors:** Kevin James Metcalf, James Lea Bevington, Sandy Lisette Rosales, Lisa Ann Burdette, Elias Valdivia, Danielle Tullman-Ercek

**Affiliations:** 1 Department of Chemical and Biomolecular Engineering, University of California, Berkeley, CA 94720 USA; 2 Department of Nutritional Science and Toxicology, University of California, Berkeley, CA 94720 USA; 3 Department of Plant and Microbial Biology, University of California, Berkeley, CA 94720 USA; 4 Department of Chemical and Biological Engineering, Northwestern University, Evanston, IL 60208 USA; 5 Department of Biomedical Engineering, Northwestern University, Evanston, IL 60208 USA

**Keywords:** Protein secretion, T3SS, Protein folding

## Abstract

**Background:**

Bacterial production of natively folded heterologous proteins by secretion to the extracellular space can improve protein production by simplifying purification and enabling continuous processing. In a typical bacterial protein production process, the protein of interest accumulates in the cytoplasm of the cell, requiring cellular lysis and extensive purification to separate the desired protein from other cellular constituents. The type III secretion system of Gram-negative bacteria is used to secrete proteins from the cytosol to the extracellular space in one step, but proteins must unfold during translocation, necessitating the folding of secreted proteins in the extracellular space for an efficient production process. We evaluated type III secretion as a protein production strategy by characterizing and quantifying the extent of correct folding after secretion.

**Results:**

We probed correct folding by assaying the function after secretion of two enzymes—beta-lactamase and alkaline phosphatase—and one single-chain variable fragment of an antibody. Secreted proteins are correctly folded and functional after unfolding, secretion, and refolding in the extracellular space. Furthermore, structural and chemical features required for protein function, such as multimerization and disulfide bond formation, are evident in the secreted protein samples. Finally, the concentration of NaCl in the culture media affects the folding efficiency of secreted proteins in a protein-specific manner.

**Conclusions:**

In the extracellular space, secreted proteins are able to fold to active conformations, which entails post-translational modifications including: folding, multimerization, acquisition of metal ion cofactors, and formation of disulfide bonds. Further, different proteins have different propensities to refold in the extracellular space and are sensitive to the chemical environment in the extracellular space. Our results reveal strategies to control the secretion and correct folding of diverse target proteins during bacterial cell culture.

**Electronic supplementary material:**

The online version of this article (doi:10.1186/s12934-016-0606-4) contains supplementary material, which is available to authorized users.

## Background

Heterologous protein production is used to make protein products, such as therapeutics and industrial enzymes, and enables researchers to study proteins that would otherwise be difficult to isolate from their native source. In order for a protein to perform its function, the protein must adopt a three-dimensional structure that allows for proper function. When producing a heterologous protein, it is desired to maximize both product titer and proper folding of the protein of interest. Secretion of heterologous proteins to the extracellular space holds several advantages over intracellular production: proteins accumulate outside the cell, limiting cytotoxicity associated with intracellular accumulation; secretion serves as a first step of purification, as the cell selectively secretes proteins to the extracellular space; and lysis of the production organism is not required, enabling continuous protein production [1, 2]. Cytosolic accumulation also may result in aggregation of the protein of interest into inclusion bodies. The insoluble inclusion body is then dissolved and refolded in dilute solution in vitro, a difficult process that results in product losses [3]. Bacteria are often used as a cellular host for protein production due to their fast growth, high protein production capacity, and inexpensive culture cost. However, not all proteins are efficiently secreted by bacteria [1].

The type III secretion system (T3SS) is a protein secretion machine found in Gram-negative pathogenic bacteria. This multimeric heteroprotein structure is characterized by a long passageway that is 2–3 nm in internal diameter, termed the needle [4]. Given the diameter of a typical folded protein, considerable unfolding of the protein is required in order to fit through the needle. It is hypothesized that only secondary structures could exist in the secreted protein during translocation. Indeed, cryo-electron microscopy of secretion suggests that proteins are fully linearized before being ejected into the extracellular space [5]. Proteins secreted by a T3SS have been previously shown to adopt a native conformation after secretion, both in the extracellular space and when delivered to the cytoplasm of a neighboring cell [6–8].

The constraints of this system present a unique condition for protein folding. Proteins are secreted by the T3SS at a rate of 10^3^–10^4^ amino acids per second per apparatus [9, 10] (about 1–10 proteins per second) and must be unfolded in order to pass through the T3SS [5]. Thus, proteins are released rapidly into the extracellular space in an unfolded and extended confirmation, in contrast to the mechanism of co-translational folding. Additionally, the extracellular space has a much lower macromolecule concentration compared to inside the cell [11]. As a result, protein folding post-secretion may resemble in vitro refolding in dilute solution. By capitalizing on this feature of protein folding and coupling production with secretion, this T3SS-based approach may hold advantages over industrial approaches that are based on inclusion body formation that requires a separate refolding step [12].

In this study, we tested the biochemical requirements for protein function to understand protein folding following secretion by the T3SS. We used protein function (e.g., enzymatic activity or antigen binding) as a proxy for folding. We investigated the ability two enzymes (beta-lactamase and alkaline phosphatase) and one single-chain variable fragment (scFv) of an antibody to adopt an active conformation after secretion. We found in all cases that protein secretion to the extracellular space allows the production of functional, correctly folded protein product. Moreover, we found that the concentration of sodium chloride in the culture medium could affect both secreted protein titer and the fraction of secreted proteins that are correctly folded, allowing for simultaneous optimization of both protein titer and folding.

## Results

### Secreted proteins are functional after secretion

**Fig. 1:**
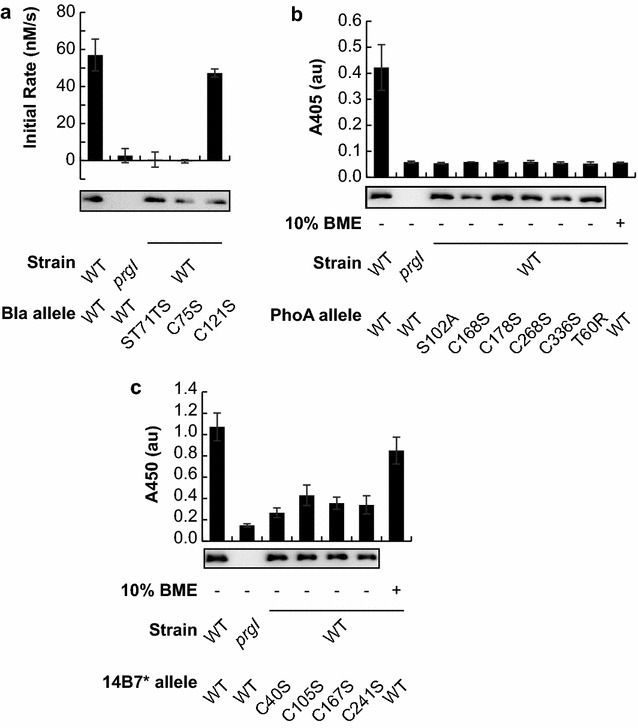
Secreted proteins adopt functional conformations. Activity or ELISA signal is given for samples analyzed from the culture supernatant. Genetic modifications described are with respect to the mature native protein sequence of the POI in the fusion. All proteins are of the format SptP-POI-2xFLAG-6xHIS. Results are plotted for the POIs. **a** Beta-lactamase (Bla). **b** Alkaline phosphatase (PhoA). **c** Single chain variable fragment against anthrax protective antigen (14B7*). The mean is plotted from three biological replicate experiments and the error bars represent one standard deviation. Western blots are representative of the samples analyzed in the functional assays

Beta-lactamase (EC:3.5.2.6, class A) is a monomeric enzyme that forms one intrachain disulfide bond, but is not required for activity [13]. No cofactors are required for activity [14]. We previously reported that the enzyme beta-lactamase adopts a catalytically active conformation after secretion by the T3SS [6]. We confirmed that beta-lactamase was indeed active in the extracellular space after secretion by the T3SS, and found that enzymatic activity in the extracellular space was both enzyme- and secretion-dependent (Fig. 1a). No secretion or activity was detected when secretion was prevented by deletion of the *prgI* gene, which codes for an essential component of the SPI-1 T3SS [15]. No activity was detected when the catalytic site of the enzyme was knocked out (ST71TS) [16], though the protein was still secreted. These results indicate that detected activity in the extracellular space was due to a catalytically active beta-lactamase. We mutated the two cysteine residues in beta-lactamase to serine to prevent disulfide bond formation. Both mutant enzymes were secreted, and the C121S mutation resulted in a catalytic activity similar to the wild type. Interestingly, the C75S mutation was not catalytically active, in contrast to previous reports in the literature [13]. Differences in N- and C-terminal modification may explain this difference—our secreted beta-lactamase bears a substantial N-terminal secretion signal and C-terminal epitopes that may affect the essentiality of Cys75.

The enzyme alkaline phosphatase (EC 3.1.3.1, isozyme 1) requires the acquisition of two Zn^2+^ and one Mg^2+^ cofactors, dimerization, and the formation of two intrachain disulfide bonds for catalytic activity [17]. Catalytic activity in the extracellular space was detected, indicating that alkaline phosphatase folded and satisfied all structural requirements for activity in the extracellular space (Fig. 1b). No secretion or activity was detected when secretion was prevented by deletion of the *prgI* gene, and no activity was detected when the catalytic site of is knocked out (S102A) [18], though the protein was still secreted. Systematic mutation of each of the four cysteines to serine to prevent disulfide bond formation resulted in secreted but catalytically inactive enzyme. In addition, no activity was detected after chemical reduction of wild type alkaline phosphatase with 10% v/v 2-mercaptoethanol. Further, no activity was detected in a monomeric alkaline phosphatase mutant (T60R) [19], though this mutant protein was still secreted. Together, these data indicate that alkaline phosphatase folds into a catalytically active conformation, including the correct formation of disulfide bonds, in the extracellular space after secretion by the SPI-1 T3SS.

A single-chain variable fragment (scFv) of an antibody is a monomeric protein that forms, but does not necessarily require, two intrachain disulfide bonds. 14B7* is an scFv of a mouse IgG antibody that binds to the protective antigen (PA) of the anthrax toxin [20, 21]. Binding of secreted 14B7* to PA was detected by enzyme-linked immunosorbent assay (ELISA) (Fig. 1c). No secretion or activity was detected when secretion is prevented by deletion of the *prgI* gene. Systematic mutation of each of the four cysteines to serine to prevent disulfide bond formation resulted in secretion and antigen binding, though each of the four mutants exhibited lower binding than wild type. Chemical reduction of the wild type 14B7* secreted sample with 10% 2-mercaptoethanol did not affect binding activity, suggesting that disulfide bonds are not essential for binding activity after post-secretion folding.

### Secreted proteins form disulfide bonds

**Fig. 2:**
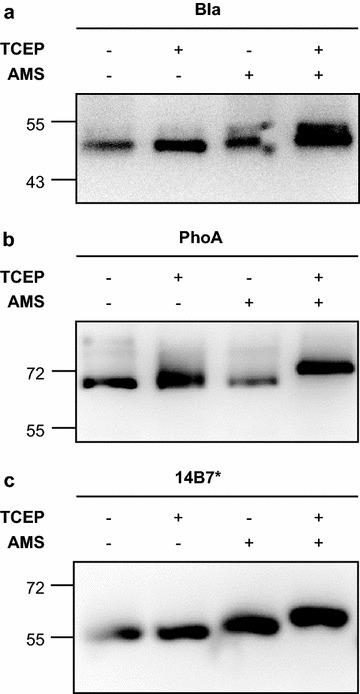
Western blots of secreted fusion protein samples subjected to the selective alkylation procedure separated by SDS-PAGE. All proteins are of the format SptP-POI-2xFLAG-6xHIS. Representative images are presented from a western blot for the POIs. **a** Bla. **b** PhoA. **c** 14B7*

The presence of disulfide bonds in secreted proteins was confirmed by selective cysteine alkylation with the reagent 4′-acetamido-4′-maleimidylstilbene-2,2′-disulfonic acid (AMS). AMS selectively adds to free thiols, adding ~500 Da of mass with each addition. It will not covalently modify cysteines that participate in a disulfide bond. Reduction of the protein sample with tris(2-carboxyethyl)phosphine (TCEP) will reduce disulfide bonds and convert all cysteines to the free thiol form. Thus, we can observe the disulfide bond state of a protein by detecting changes in molecular weight resulting from redox-dependent protein modification by AMS [22]. Greater cysteine modification will result in a protein that migrates more slowly in a denaturing polyacrylamide gel. For all proteins tested, the N-terminal SptP secretion signal sequence contains a cysteine residue at position 112 that is not expected to participate in a disulfide bond and is thus a free thiol. Indeed, a shift in migration was detected when all proteins are modified with AMS without TCEP pretreatment, indicating that the cysteine in the SptP secretion signal sequence is modified (Fig. 2, lane 3).

Disulfide bonds were detected in beta-lactamase (Fig. 2a). This protein contains one intrachain disulfide bond in the native protein, giving a total of three cysteine residues in the fusion protein. An increase in apparent molecular weight was observed when the protein was modified with AMS after TCEP pretreatment, indicating that the protein contained a disulfide bond in the extracellular space. Disulfide bonds were also detected in both alkaline phosphatase and the 14B7* scFv (Fig. 2b, c). Both of these proteins contain two intrachain disulfide bonds in the native protein, giving a total of five cysteine residues in the fusion protein. When the sample was pretreated with TCEP before modification with AMS, a further increase in apparent molecular weight was observed, indicating that disulfide bonds were present in the secreted protein.

### Specific activity of secreted enzymes is affected by salt concentration in growth medium

Activity of the secreted enzymes was compared with enzyme purified from the cytosol. While activity of the secreted enzymes was detected as shown in Fig. 1, it was not clear what fraction of the secreted enzymes were active. We define the parameter *f*_*fold*_ as the fraction of functional secreted protein, relative to the same protein fusion purified from the cytoplasm. Briefly, we assume that secreted enzymes that are folded are also active and catalyze reactions with rate *k*_*cat*_, while misfolded secreted enzymes do not contribute to catalysis. The sample thus catalyzes reaction with an apparent rate constant, $k_{cat}^{app}$, that is less than or equal to *k*_*cat*_ (see Additional file 1 section for a thorough description of the *f*_*fold*_ parameter and the apparent rate constant $k_{cat}^{app}$).

Beta-lactamase and alkaline phosphatase were purified from the cytosol and the enzyme concentration, [E]_T_, and the kinetic parameters *K*_M_, $k_{cat}^{app}$, and *V*_*max*_ were calculated for each sample. In addition, the same kinetic parameters were calculated for secreted enzyme (Table 1). This analysis was first performed in standard production media (Lysogeny Broth, Lennox; 5 g L^−1^ NaCl) [6].

**Table 1:** Analysis of refolding efficiency of secreted enzyme in the culture supernatant, relative to purified, soluble cellular enzyme

	Secreted		Purified		
Protein	$k_{cat}^{app}$ (s^−1^)	*K*_M_ (µM)	$k_{cat}^{app}$ (s^−1^)	*K*_M_ (µM)	*f*_*fold*_
Bla	38 ± 4	28 ± 11	248 ± 11	42 ± 6	0.15 ± 0.02
PhoA	22 ± 2	100 ± 40	26 ± 2	230 ± 80	0.85 ± 0.10

Uncertainty is given as the standard error and is propagated for the *f*_*fold*_ calculation. All experiments were performed three times in biological replicate. Parameters for secreted samples were calculated for samples generated in LB media with 5 g L^−1^ NaCl

The fraction of secreted beta-lactamase enzymes that are active was 15 ± 2%, relative to the purified form. The kinetic parameters *K*_M_ and $k_{cat}^{app}$ of the purified beta-lactamase fusion compared well with published values for the wild type enzyme for the nitrocefin substrate (110 µM and 900 s^−1^, respectively) [23]. No statistically significant difference in the value of *K*_M_ was found between purified and secreted beta-lactamase fusion. However, the secreted and purified forms of beta-lactamase significantly differed in the calculated apparent rate constant, $k_{cat}^{app}$ (p < 0.05), suggesting a folding defect in the secreted enzyme, relative to the enzyme purified from the cell.

The fraction of secreted alkaline phosphatase enzymes that are active was 85 ± 10%, relative to the purified form. Both kinetic parameters, *K*_M_ and $k_{cat}^{app}$, of the purified alkaline phosphatase fusion were significantly different from the published values for the wild type enzyme for the para-nitrophenyl phosphate substrate (35 ± 5 µM and 176 ± 6 s^−1^, respectively) [24]. It should be noted that these reported kinetic parameters reported by Wojciechowski and Kantrowitz were calculated for reactions performed at 25 °C in a Tris-buffered solution, while all reactions with alkaline phosphatase in this study were conducted at 37 °C in LB media, prohibiting a direct comparison of the values. No statistically significant differences in the values of *K*_M_ and $k_{cat}^{app}$ were found between purified and secreted alkaline phosphatase fusion. Thus, the activity of the alkaline phosphatase fusion studied in this work did not experience a significant folding defect after secretion, compared to protein purified from soluble cytosolic fraction. It should be noted that while the value of *K*_M_ for the secreted and purified samples was not statistically significantly different, the large difference in *K*_M_ between the samples may indicate that the folding of alkaline phosphatase is not well described by our simple two-state model (see Additional file 1 section for details on two-state model).

**Fig. 3:**
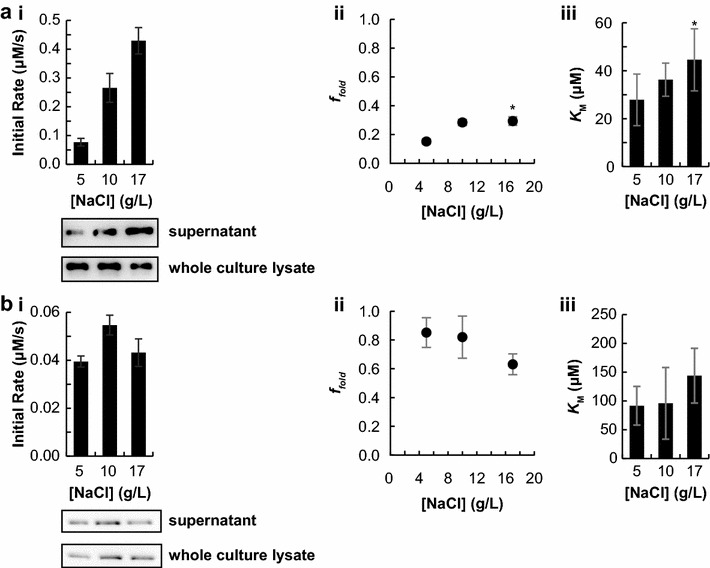
NaCl concentration in media affects secreted protein titer and folding efficiency. Both proteins studied are in the fusion format SptP-POI-2xFLAG-6xHIS. For all plots, unless specified, the mean of three biological replicates is plotted, except where noted by the symbol * to indicate two biological replicates. Error bars represent one standard deviation, unless noted. **a** *i* Plot of raw activity of secreted Bla as a function of growth media with representative western blot of analyzed samples. *ii* Plot of *f*_*fold*_ for secreted Bla as a function of growth media. *iii* Plot of *K*_M_ for secreted Bla as a function of growth media. Error bars represent the standard error. **b**. *i* Plot of raw activity of secreted PhoA as a function of growth media with representative western blot of analyzed samples. *ii* Plot of *f*_*fold*_ for secreted PhoA as a function of growth media. *iii* Plot of *K*_M_ for secreted PhoA as a function of growth media. Error bars represent the standard error

We attempted to increase the parameter *f*_*fold*_ by changing culturing conditions. By changing the components in the growth medium, we hypothesized that the folding of secreted protein could be modulated. The ionic strength of a solution is known to affect protein folding, likely through charge–charge interactions [25]. The concentration of NaCl in the growth medium was varied between 5 and 17 g L^−1^ (0.09 and 0.3 M, respectively). The activity was then calculated using activity assays and quantitative western blotting, as above. Interestingly, the activity of beta-lactamase at saturating concentrations of substrate increased with NaCl concentration in the growth medium. This effect can be attributed in part to the fact that secreted protein titer increased monotonically with increasing [NaCl] (Fig. 3a.i). However, importantly, the increased activity in Fig. 3a.i was due to both increased enzyme concentration and increased *f*_*fold*_ (Fig. 3a.ii). Further, the calculated value of *K*_M_ for secreted beta-lactamase was not significantly different between the three media conditions tested (Fig. 3a.iii).

The effect of NaCl in the growth media had an opposite effect on alkaline phosphatase. No change was observed in secreted protein titer or activity at saturating concentrations of substrate (Fig. 3b.i). The value of *f*_*fold*_ did not change at low concentrations of NaCl but decreased at the highest salt concentration (Fig. 3b.ii). The calculated value of *K*_M_ for secreted alkaline phosphatase was not significantly different between the three conditions tested (Fig. 3b.iii).

## Discussion

Pure and correctly folded protein is desired when producing a heterologous protein. Production of heterologous proteins via secretion to the extracellular space holds many advantages over intracellular accumulation: purification is simplified; cytotoxicity is alleviated; and cell lysis is not required [1, 2]. The production and secretion of heterologous proteins can be achieved using the T3SS of various Gram-negative bacteria [7–9, 26, 27]. However, the mechanism of protein secretion requires an unfolding event during translocation [5]. For this production strategy to be effective, the protein should fold into a functional conformation after secretion. This event must occur in the extracellular space, a region of the cell culture thought to be devoid of molecular chaperones that assist in protein folding. By testing for protein function, the folded state of the protein is probed, as only folded proteins are expected to be functionally active. The protein function assays are sensitive enough to give information on the folded state of the protein of interest in a heterogeneous, dilute protein mixture.

Previous studies have demonstrated the ability of heterologous proteins secreted by a T3SS to adopt active conformations [6–8]. In this study, we investigated the ability of secreted proteins with different chemical and structural requirements to adopt active conformations. The enzyme beta-lactamase folds into a catalytically active tertiary structure (Fig. 1a). The enzyme alkaline phosphatase adopts a catalytically active tertiary structure, forms a dimer and two intrachain disulfide bonds, and acquires one Mg^2+^ and two Zn^2+^ ions per chain (Fig. 1b). The scFv 14B7* folds into a tertiary structure that permits antigen binding (Fig. 1c). These data demonstrate that proteins can adopt active confirmations after secretion. Furthermore, in the extracellular space—an area thought to be devoid of folding chaperones—formation of disulfide bonds, multimerization, and acquisition of metal ion cofactors can still occur. We hypothesize that these interactions occur spontaneously in the extracellular space. It is unlikely that post-translational modifications are achieved inside the cell before secretion, owing to the limited conformations allowed by the type III secretion system lumen. The cultures are grown aerobically, and it is likely that the oxidizing environment of the culture medium allows for disulfide bond formation. It is not clear, however, what molecular species, if any, are involved in disulfide bond formation. Dissolved oxygen and/or other components of the growth media may be involved in disulfide bond formation. Further, the growth media is not chemically defined and likely contains trace metals for secreted proteins to sequester. Nonetheless, it is surprising that these proteins are able to adopt active conformations in the extracellular space after secretion.

A comparison of the specific activity of secreted and purified cellular enzyme was performed to compare the fraction of secreted proteins that adopt active conformations in the extracellular space. Secreted enzyme that is catalytically inactive will decrease the apparent rate constant, $k_{cat}^{app}$. Indeed, beta-lactamase had a lower value of $k_{cat}^{app}$ in the secreted sample, compared to the purified protein (Table 1). This suggests that only a fraction of the secreted enzyme adopts an active conformation after secretion. Extending this reasoning, the percentage of secreted beta-lactamase that is active is 15 ± 2% (Table 1). We speculate that this is due to misfolding or aggregation. Increasing the ionic strength of a solution has been shown to increase the thermodynamic stability of a folded monomeric protein [25], increasing the concentration of NaCl in the growth medium from 5 to 17 g L^−1^ increased *f*_*fold*_ of secreted beta-lactamase by almost threefold (Fig. 3a.ii). This indicates that the ability of the secreted protein to adopt an active conformation after secretion may be dependent on the chemical environment in which it folds. Secreted alkaline phosphatase had a $k_{cat}^{app}$ value similar to the purified sample, such that the percentage of folded enzyme is 85 ± 10% for the 5 g L^−1^ NaCl sample (Table 1). This indicates that the fraction of folding for secreted alkaline phosphatase is similar to the folding when the enzyme is purified from the soluble cellular fraction following cytosolic expression. Increasing the concentration of NaCl from 5 to 10 g L^−1^ in the culture medium did not increase the value of *f*_*fold*_ (Fig. 3b.ii). However, the value of *f*_*fold*_ decreased at the highest media NaCl concentration. This effect could be due to increased charge screening at high solution ionic strength, as charge–charge interactions are known to be important in dimerization of alkaline phosphatase [19, 28].

Understanding protein folding after secretion is important for improving protein production. Large-scale protein production often involves production of the protein of interest in inclusion bodies, which are then solubilized and refolded. Parallels exist between protein folding in the extracellular space and inclusion body solubilization followed by protein refolding in vitro. In an in vitro refolding procedure, the inclusion body is solubilized in a high concentration solution of a chaotrope, such as guanidinium chloride. This also unfolds the proteins in the inclusion body. The solution is then diluted to allow for proteins to fold into a native conformation. Dilution decreases the concentration of protein in solution in addition to decreasing the concentration of chaotrope. The lower concentration of protein in solution improves protein refolding, as each chain is less likely to form interchain aggregates. Secretion of protein to the extracellular space mimics this process, as the extracellular space also has a lower protein concentration, relative to inside the bacterial cell. The needle structure of the T3SS extends ~50 nm from the outer membrane [29], potentially beyond any extracellular cellular structures, such as the lipopolysaccharide layer. Thus, we speculate that proteins secreted by the T3SS to the extracellular space will experience a folding environment that, as in in vitro refolding, can be tailored to increase the folding of the secreted protein. In addition to allowing the facile production of correctly folded heterologous protein in the supernatant of bacterial culture, protein secretion by the T3SS offers a unique condition to study protein folding. Refolding of proteins after secretion may access different folding trajectories than found in co-translational or in vitro folding conditions. Given the rate and directionality of secretion, proteins may fold in a unique vectorial folding pathway. Further, the unfolded conformations that are accessible to the protein during secretion are likely constrained, which in turn may change the conformations that are accessible after secretion. We anticipate that this system will provide a unique folding environment for future study.

## Conclusions

Production of heterologous proteins in bacteria is improved using a secretion strategy. In this study, the T3SS of *Salmonella enterica* was used to secrete proteins to the extracellular space. A folded protein product in the extracellular space is desired, but the T3SS secretion mechanism requires proteins to be unfolded during translocation. We characterized folding of secreted proteins by quantifying protein function, either by enzymatic activity or antigen binding. Indeed, the three secreted proteins in this study adopt a functional confirmations after secretion to the extracellular space. However, only a fraction of secreted proteins are functional, between 10 and 85%. Media composition was used to modify the chemical environment in the extracellular space. Increasing the concentration of NaCl in the culture medium had a protein-specific effect on the ability of a secreted protein to adopt an active conformation in the extracellular space. Further understanding of how chemical and genetic variables control the folding of secreted proteins will be important for process development of bacterial strains for production of secreted proteins. An improved production process achieves secretion a protein at high titer to the extracellular space and that spontaneously adopts a functional conformation. This would simplify purification and not require refolding unit operations.

## Methods

### Strains and growth conditions

All *S. enterica* experiments used derivatives of the SL1344 strain [30]. The *prgI* deletion strain was described by Metcalf et al. [6]. All strains were grown from colonies from fresh transformations or fresh streaks from frozen stock in lysogeny broth (LB-Lennox, LB-L) (10 g L^−1^ tryptone, 5 g L^−1^ yeast extract, and 5 g L^−1^ NaCl) (VWR #EM1.00547.5007) with appropriate antibiotics (34 µg mL^−1^ chloramphenicol and/or 50 µg mL^−1^ kanamycin) for 12–16 h at 37 °C and 225 rpm in an orbital shaker overnight. Overnight cultures were subcultured into fresh LB-L media supplemented with 100 µM isopropyl β-d-1-thiogalactopyranoside (IPTG) and the appropriate antibiotics. All culturing steps were performed in 24-well blocks (Axygen).

*Salmonella enterica* strains were transformed with the plasmids listed in Additional file 1: Table S1 using electroporation. All experiments were performed using a two-plasmid system, with the upregulation vector (P_lacUV5_ *hilA*), as reported by Metcalf et al. [6], in addition to the export vector that carried the gene coding for the protein of interest [26]. Controlled overexpression of *hilA* allows for controlled expression of genes coding for both the secretion apparatus and the protein of interest [6].

### DNA manipulations

PCR was performed with Pfu DNA polymerase and the primers listed in Additional file 1: Table S2. Restriction enzymes and ligase (NEB) were used according to the manufacturer’s instructions. For all cloning, *E. coli* DH10B cells were used. Mutations to *bla* and *phoA* are specified with respect to the full-length mature wild type protein. Mutations to 14b7* are specified with respect to the order of cysteines, from amino to carboxy terminus, in the antibody fusion gene and does not include the cysteine in the N-terminal SptP signal sequence. The *bla* gene was amplified from the plasmid pTrc99A [31]. The *phoA* gene was amplified from *E. coli* MG1655. The 14b7* gene was amplified from the plasmid pFLAG-APEx 14B7* (gift from the Georgiou lab).

### Protein separation and western blotting

Samples were separated by SDS-PAGE. Proteins were transferred to a polyvinylidene fluoride (Millipore) membrane for chemiluminescence detection, using the TransBlot SD unit (Bio-Rad). Membranes were interrogated with Mouse anti-FLAG antibodies per manufacturer’s instructions (Sigma). A secondary labeling step was carried out with Goat anti-Mouse IgG (H + L), HRP conjugate antibodies, per manufacturer’s instructions (Thermo). Bands were visualized with west-pico or west-femto chemiluminescent substrate (Thermo) and imaged with a ChemiDoc XRS + unit (Bio-Rad).

### Protein purification

Culture homogenate was purified using a His GraviTrap column (GE Healthcare # 11-0033-99). Eluted protein sample was separated by SDS-PAGE, stained with Coomassie G-250, and quantified using densitometry relative to a bovine serum albumin standard (Thermo). Purified protein samples were diluted in LB-L and stored at 4 °C for enzyme activity assays.

### Protein quantification

Supernatant samples were harvested 8 h after subculture. Supernatant samples were harvested from the cell culture by two sequential centrifugation steps of 2272×*g* for 10 min, 4× Laemmli buffer was added to each sample for a final concentration of Laemmli buffer of 1×, and boiled for 95 °C for 5 min. Purified protein samples were used to create four standards that were included with each blot to construct a standard curve. Samples were separated by SDS-PAGE and transferred to a polyvinylidene fluoride (Millipore). A linear least-squares regression of the standard samples were used to calculate the concentration of each supernatant sample.

### Beta-lactamase activity assay

Samples were grown overnight in LB-L media, then subcultured 1:100 in LB-L media and grown for 8 h at 37 °C and 225 rpm. The cultures were pelleted by one centrifugation step of 2272×*g* for 10 min and the supernatant was passed through a 0.45 μm filter. Samples were then subjected to a nitrocefin hydrolysis assay, per the substrate vendor (Sigma). 100 μL of reaction buffer (0.1 M phosphate, 1 mM ethylenediaminetetraacetic acid, 50 g mL^−1^ nitrocefin (EMD Millipore), 0.5% dimethyl sulfoxide, pH 7) was mixed with 10 μL culture supernatant and the absorbance at 486 nm was observed over time. The reaction was performed in a disposable UV-Transparent cuvette (BrandTech, part# 759215) without stirring at 37 °C. The absorbance at 486 nm was measured every 5 s for 60 s with a UV–Vis spectrophotometer (Nanodrop, part# 2000c). The reaction was linear for the first 40 s of all reactions tested and the change in the absorbance as a function of time was determined using a linear least-squares fit. The initial reaction velocity was calculated using an extinction coefficient of 20,500 M^−1^ cm^−1^, as specified by the vendor. The experiment was performed on different days in biological triplicate. Error bars represent one standard deviation.

### Alkaline phosphatase activity assay

Samples were grown overnight in LB-L media, then subcultured 1:100 in LB-L media and grown for 8 h at 37 °C and 225 rpm. The cultures were pelleted by one centrifugation step of 2272×*g* for 10 min and the supernatant was passed through a 0.45 μm filter. Samples were then subjected to a para-nitrophenyl phosphate assay, modified from Glasgow et al. [32]. Briefly, supernatant samples were mixed with the appropriate volume of 1 M Tris (base), pH 8.0 and 0.4 w/w% para-nitrophenyl phosphate. For endpoint assays, multiple equal-volume reactions were performed simultaneously in a 96-well microtiter plate and incubated at 37 °C without shaking for at least 1 h. The absorbance at 405 nm of each well was measured using a Synergy HTX Multi-Mode Reader spectrophotometer (Bio-Tek). For kinetic assays, the reaction was performed in a disposible UV-Transparent cuvette (BrandTech, part# 759215) without stirring at 37 °C. The absorbance at 405 nm was measured every 5 s for 60 s with a UV–Vis spectrophotometer (Nanodrop, part# 2000c). The reaction was linear for the first 40 s of all reactions tested and the change in the absorbance as a function of time was determined using a linear least-squares fit. The initial reaction velocity was calculated using an extinction coefficient of 18,000 M^−1^ cm^−1^, as specified by the vendor. The experiment was performed on different days in biological triplicate. Error bars represent one standard deviation.

### Enzyme-linked immunosorbent assay (ELISA)

Samples were grown overnight in LB-L media, then subcultured 1:100 in LB-L media and grown for 8 h at 37 °C and 225 rpm. The cultures were pelleted by one centrifugation step of 2272×*g* for 10 min and the supernatant was passed through a 0.45 μm filter. The wells of a 96-well microtiter plate (Santa Cruz Biotechnology, Inc., part# sc-204463) was coated with 100 µL of a solution of 4 µg mL^−1^ protective antigen (PA) of the anthrax toxin (List Biological Laboratories, part# 171E) in 5 mM HEPES, 50 mM NaCl, pH 7.5 covered at 4 °C overnight. Liquid was removed by inversion and wells were incubated with a 200 µL blocking solution (200 µL 2% milk, 0.05% TBST) at room temperature for 1 h. Liquid was removed by inversion and 100 µL filtered culture supernatant was added to the wells. Samples were incubated at room temperature for 2 h. Liquid was removed by inversion and wells were rinsed with 0.05% TBST three times. Next, 100 µL of primary labeling solution (1:10,000 dilution of Mouse anti-FLAG antibody (Sigma) diluted in 0.05% TBST) was added to the wells and incubated 1 h at room temperature. Liquid was removed by inversion and wells were rinsed with 0.05% TBST five times. Then, 100 µL of secondary labeling solution (1:5000 dilution of Goat anti-Mouse IgG (H + L), HRP conjugate antibodies (Thermo) diluted in 0.05% TBST) was added to the wells and incubated 1 h at room temperature. Liquid was removed by inversion and wells were rinsed with 0.05% TBST five times. 100 µL of 3,3′,5,5′-Tetramethylbenzidine (TMB) Liquid Substrate (Thermo) was added to the wells and incubated for approximately 20 min at room temperature. The reaction was quenched with 100 µL 2 M H_2_SO_4_. Absorbance at 450 nm was measured using a Synergy HTX Multi-Mode Reader spectrophotometer (Bio-Tek).

### Cysteine alkylation

Filtered supernatant samples were precipitated with trichloroacetic acid (20% w/w final concentration) overnight at 4 °C, washed twice with cold acetone, and dried by heating. Dried samples were resuspended in 50 µL resuspension buffer (1 M Tris (base), pH 7.5, 3% w/w sodium dodecyl sulfate). Resuspension buffer was supplemented with 10 mM tris(2-carboxyethyl)phosphine (TCEP) for reduced samples, as necessary. Samples were incubated at room temperature for 10 min. Next, 8.8 µL of 100 mM 4-acetamido-4′-maleimidylstilbene-2,2′-disulfonic acid (AMS) was added to samples (final concentration is 15 mM), as necessary. Samples to which AMS was not added were diluted with equal volume distilled water. Samples were incubated at room temperature for 2 h, protected from light. Samples were then mixed with Laemmli buffer, boiled, and separated by SDS-PAGE, and bands were detected by a western blot, as specified above.

### Error estimation of Michaelis–Menten model

Error estimation was conducted using Matlab (Mathworks, R2014a). Biological replicates were treated as independent samples. Three independent replicates were then analyzed for both purified and secreted samples. Least-squares minimization was used to fit a modified Michaelis-Menten model (Eq. 1) to the measured values and determine the parameters $k_{cat}^{app}$ and *K*_M_.

1 $$ \frac{{V_{0} }}{{\left[ E \right]_{T} }} = \frac{{k_{cat}^{app} \cdot \left[ S \right]}}{{K_{M} + \left[ S \right]}} $$

where *V*_0_ is the initial reaction rate, [E]_T_ is the total enzyme concentration, [S] is the substrate concentration, and $k_{cat}^{app}$ and *K*_M_ are fitting parameters. To compare the parameters from different treatments, a *t* test (Eq. 2) was used where the degrees of freedom was determined by the pooled sample size.

2 $$ t = \frac{{\beta_{1} - \beta_{2} }}{{\sqrt {Se_{1}^{2} - Se_{2}^{2} } }} $$

where *t* is the *t* statistic, *β* is the fitting parameter of interest (i.e., $k_{cat}^{app}$ and *K*_M_), and *Se* is the standard error calculated from the nonlinear regression. A p value of 0.05 was used to define significance.

## Additional file

**Additional file 1. Supplemental calculations and tables.**

## Abbreviations

AMS: 4′-acetamido-4′-maleimidylstilbene-2,2′-disulfonic acid
ELISA: enzyme-linked immunosorbent assay
LB: lysogeny broth
PA: protective antigen of anthrax toxin
scFv: single-chain variable fragment
TCEP: tris(2-carboxyethyl)phosphine
T3SS: type III secretion system
