# Supplementary material for: Proteins adopt functionally active conformations after type III secretion
Source: Microb Cell Fact. 2016 Dec 23;15:213. doi: 10.1186/s12934-016-0606-4 (PMC5180411; doi:10.1186/s12934-016-0606-4)
Supplement: Supplementary file 1 — Additional file 1. Supplemental calculations and tables. [file 12934_2016_606_MOESM1_ESM.docx]

# Supplemental Information:

**Title:**

Proteins adopt functionally active conformations after type III secretion

**Authors:**

Kevin James Metcalf^1ϟ^, James Lea Bevington^1^, Sandy Lisette Rosales^2^, Lisa Ann Burdette^1,3^, Elias Valdivia^4^, Danielle Tullman-Ercek^3^*

**Affiliations:**

1. Department of Chemical and Biomolecular Engineering, University of California, Berkeley, CA 94720, USA 2. Department of Nutritional Science and Toxicology, University of California, Berkeley, CA 94720, USA 3. Department of Chemical and Biological Engineering, Northwestern University, Evanston, IL 60208, USA 4. Department of Plant and Microbial Biology, University of California, Berkeley, CA 94720, USA

* To whom correspondence should be addressed. E-mail: [ercek@northwestern.edu](mailto:ercek@northwestern.edu).

ϟ Present address: Department of Biomedical Engineering, Northwestern University, Evanston, IL 60208, USA

**Calculation of *f_fold_*.** First, we assume that secreted proteins exist in two states: state 1) catalytically active; and state 2) catalytically inactive. Yet, the quantitative western blot assay experimentally determines the total secreted protein concentration, [E]_T_, which includes both active (state 1) and inactive (state 2) forms of the secreted enzyme (Eq. S1):

$\left[\text{E}\right]_{\text{T}}=\left[\text{E}\right]_{\text{active}}+\left[\text{E}\right]_{\text{inactive}}$ (S1)

where [E]_active_ is the concentration of secreted enzyme that is catalytically active (state 1) and [E]_inactive_ is the concentration of secreted enzyme that is not catalytically active (state 2). We then define a parameter, *f_fold_*, that is the fraction of secreted enzyme that is active:

$f_{fold}=\frac{\left[\text{E}\right]_{\text{active}}}{\left[\text{E}\right]_{\text{T}}}$ (S2)

In this two state model, secreted enzymes in state 1 catalyze reactions with the rate constant *k_cat_*, while secreted enzymes in state 2 do not catalyze reactions, and thus have a rate constant,

$k_{cat}^{inactive}$, equal to zero. We then apply this assumption to a modified Michaelis-Menten equation, yielding Eq. S3:

$\frac{dP}{dt}=\frac{k_{cat}\left[\text{E}\right]_{\text{active}}\left[S\right]}{K_{M}+\left[S\right]}=\frac{k_{cat}\left[\text{E}\right]_{\text{T}}\left[S\right]}{K_{M}+\left[S\right]}f_{fold}$ (S3)

We then define an apparent rate constant, $k_{cat}^{app}$, that is the observed reaction rate:

$k_{cat}^{app}=f_{fold}k_{cat}$ (S4)

We combine Eqs. S3 and S4 to yield Eq. S5:

$\frac{dP}{dt}=\frac{k_{cat}^{app}\left[\text{E}\right]_{\text{T}}\left[S\right]}{K_{M}+\left[S\right]}$ (S5)

Thus, we calculate the value of *f_fold_* by taking the ratio of the values of the fitted parameters $k_{cat}^{app}$ and *k_cat_*. This allows for calculation of the fraction of total secreted protein that is folded, using protein purified from the cytosol as a reference state that we assume to be exclusively in state 1.

**Table S1.** Plasmids used in this study.

| **Plasmid name** | **ORFs under inducible control** | | **ORI** | **ab^R^** | **Reference** |
| --- | --- | --- | --- | --- | --- |
| P_lacUV5_ *hilA* | *hilA* |  | p15a | kan | Metcalf, et al., 2014 |
| P_sicA_ *bla* | *sicP* | *sptP-bla-2xF-6xH* | colE1 | cam | Metcalf, et al., 2014 |
| P_sicA_ *bla^ST71TS^* | *sicP* | *sptP-bla^ST71TS^-2xF-6xH* | colE1 | cam | This study |
| P_sicA_ *bla^C75S^* | *sicP* | *sptP-bla^C75S^-2xF-6xH* | colE1 | cam | This study |
| P_sicA_ *bla^C121S^* | *sicP* | *sptP-bla^C121S^-2xF-6xH* | colE1 | cam | This study |
| P_sicA_ *phoA* | *sicP* | *sptP-phoA-2xF-6xH* | colE1 | cam | This study |
| P_sicA_ *phoA^S102A^* | *sicP* | *sptP-phoA^S102A^-2xF-6xH* | colE1 | cam | This study |
| P_sicA_ *phoA^C168S^* | *sicP* | *sptP-phoA^C168S^-2xF-6xH* | colE1 | cam | This study |
| P_sicA_ *phoA^C178S^* | *sicP* | *sptP-phoA^C178S^-2xF-6xH* | colE1 | cam | This study |
| P_sicA_ *phoA^C286S^* | *sicP* | *sptP-phoA^C286S^-2xF-6xH* | colE1 | cam | This study |
| P_sicA_ *phoA^C336S^* | *sicP* | *sptP-phoA^C336S^-2xF-6xH* | colE1 | cam | This study |
| P_sicA_ *phoA^T60R^* | *sicP* | *sptP-phoA^T60R^-2xF-6xH* | colE1 | cam | This study |
| P_sicA_ *14B7** | *sicP* | *sptP-14B7*-2xF-6xH* | colE1 | cam | This study |
| P_sicA_ *14B7*^C40S^* | *sicP* | *sptP-14B7*^C40S^-2xF-6xH* | colE1 | cam | This study |
| P_sicA_ *14B7*^C105S^* | *sicP* | *sptP-14B7*^C105S^-2xF-6xH* | colE1 | cam | This study |
| P_sicA_ *14B7*^C167S^* | *sicP* | *sptP-14B7*^C167S^-2xF-6xH* | colE1 | cam | This study |
| P_sicA_ *14B7*^C241S^* | *sicP* | *sptP-14B7*^C241S^-2xF-6xH* | colE1 | cam | This study |

**Table S2.** Primers used in this study. For each pair, the top row is the forward primer and the bottom row is the reverse primer.

| **Sequence** | **Used for the construction of:** | |
| --- | --- | --- |
| GAACGTTTTCCAATGATGACCTCTTTTAAAGTTCTGCTATG | | *bla^ST71TS^* mutant |
| CATAGCAGAACTTTAAAAGAGGTCATCATTGGAAAACGTTC | |
| CTTTTAAAGTTCTGCTAAGCGGCGCGGTATTATCCCG | | *bla^C75S^* mutant |
| CGGGATAATACCGCGCCGCTTAGCAGAACTTTAAAAG | |
| GACAGTAAGAGAATTAAGCAGTGCTGCCATAAC | | *bla^C121S^* mutant |
| GTTATGGCAGCACTGCTTAATTCTCTTACTGTC | |
| attaggtctcaGCTTCGGACACCAGAAATGCCTG | | *P_sic_ sicP sptP-phoA-2xF-6xH* plasmid |
| attaggtctcaCGCTTTTCAGCCCCAGAGCGG | |
| CTACGTCACCGACGCGGCTGCATCAG | | *phoA^S102A^ mutant* |
| CTGATGCAGCCGCGTCGGTGACGTAG | |
| GACCTCGCGCAAAAGCTACGGTCCGAG | | *phoA^C168S^ mutant* |
| CTCGGACCGTAGCTTTTGCGCGAGGTC | |
| GCGACCAGTGAAAAAAGCCCGGGTAACGCTCTG | | *phoA^C178S^ mutant* |
| CAGAGCGTTACCCGGGCTTTTTTCACTGGTCGC | |
| GCCCGCAGTCACCAGCACGCCAAATCCGC | | *phoA^C286S^ mutant* |
| GCGGATTTGGCGTGCTGGTGACTGCGGGC | |
| CATGCTGCGAATCCTAGCGGGCAAATTGGCGAG | | *phoA^C336S^ mutant* |
| CTCGCCAATTTGCCCGCTAGGATTCGCAGCATG | |
| GGGGACTCGGAAATTCGCGCCGCACGTAATTATG | | *phoA^T60R^ mutant* |
| CATAATTACGTGCGGCGCGAATTTCCGAGTCCCC | |
| aggtctcaGCTTGAGGCCCAGCCGGCCATG | | *P_sic_ sicP sptP-14B7*-2xF-6xH* plasmid |
| aggtctcaCGCTTGCGGCCGCGAATTCGG | |
| GAGTCACCATCAGTAGCAGGGCAAGTCA | | *14B7*^C40S^ mutant* |
| CTGACTTGCCCTGCTACTGATGGTGACTC | |
| GATATTGGCACTTACTTTAGCCAACAGGGTAATACG | | *14B7*^C105S^ mutant* |
| CGTATTACCCTGTTGGCTAAAGTAAGTGCCAATATC | |
| CTCAGTGAAGATTTCCAGCAAAGATTCTGGCTAC | | *14B7*^C167S^ mutant* |
| GTAGCCAGAATCTTTGCTGGAAATCTTCACTGAG | |
| GCGGTCTATTTCAGTGCAAGGTCGGG | | *14B7*^C241S^ mutant* |
| CCCGACCTTGCACTGAAATAGACCGC | |
